# Burning mouth syndrome: controversial place as a symptom of Oro-dental pathology

**Published:** 2015

**Authors:** EC Coculescu, G Manole, BI Coculescu, VL Purcărea

**Affiliations:** * Department of Oral Medicine, Faculty of Dental Medicine, “Carol Davila” University of Medicine and Pharmacy, Bucharest, Romania; ** Faculty of Medicine, “Titu Maiorescu” University, Bucharest, Romania; *** Faculty of Medicine, “Carol Davila” University of Medicine and Pharmacy, Bucharest, Romania

**Keywords:** burning mouth syndrome, orofacial pain, neuropathic pain

## Abstract

As defined by WHO experts, disease involves a change of the physical, mental and social welfare, generating chronic stress condition if unresolved. One of the symptoms almost constantly found in any condition is pain. This feeling manifests differently depending on the subjective perception. The burning mouth syndrome (BMS) is included in such a framework. The BMS is considered as one of the characteristic states of chronic stress syndromes associated with nonspecific clinical manifestations and requires special medical attention in terms of assessing and treating the condition. However, the insufficient knowledge of its etiopathogenic mechanisms requires comprehensive research undertaken on such a subject.

## The importance of pain in general pathology

The concept of pain has evolved in the twenty-first century from that of a one-dimensional feeling to the multidimensional experience, including the sensory-discriminative qualities, cognitive, motivational and affective. Addressing such a vision, the Taxonomy Committee of the International Association for the Study of Pain (IASD) defined pain as “an unpleasant sensory and emotional experience associated with actual or potential tissue damage” [**1**].

As a symptom, pain is perceived as an unpleasant sensation, localized in a region of the body, reported as such by the patient. 

The clinical value of pain establishes the positive diagnosis results from its characters (i.e., stabbing, burning, compression etc.). Beyond the physical characteristics that define it, associated pain has a psychological component. Moreover, any moderate or greater pain is accompanied by anxiety and concern for the patient to remove it or limit soreness. Arguments allow the admission of a duality pain: symptom - signal and feeling - emotion [**2**].

The duality signal of type pain - symptoms pathogenesis involves:

a) the value of a signal conveying “information” which notes the existence of a potentially disruptive imbalance in the body’s homeostasis;

b) represents the meaning and subjective clinical expression of suffering that the body receives from the aggressive action of a noxious agent on it.

The duality of this type of pain gives a defense value [**3**].

Another important character of the pain from a semiotic point of view is the topography of pain, allowing its location in the mouth (oral).

A number of manifestations of local and general conditions may happen in the mouth, which are possible only if located in this area. The existence of such oral pathology, regardless of etiology, can occur almost constantly as pain in this anatomical and functional segment of the body. For this reason, seeking to broaden the scope of knowledge, dentists have to acquire knowledge in the general medical practice domain. Burning mouth syndrome (BMS) enrolls between types of pain, manifested in the mouth, being classified as a syndrome or disease by other authors as border, interdisciplinary, among different medical specialties closely related to the actual Dentistry.

Based on data recorded in medical literature, BMS was first described in the nineteenth century. Later, in the early twentieth century, burning mouth syndrome was characterized by Butlin and Oppenheim as glossodynia as the main headquarters of pain in most patients is the tongue [**4**-**7**]. In later years, BMS has been referred to as glossopyrosis, oral dysesthesia, sore tongue, stomatopyrosis, and stomatodynia [**4**,**6**,**8**,**9**].

The International Society first classified BMS as a distinct disorder for the Study of Headache (International Headache Society - IHS) in 2004 [**6**].

Multiple names that tried to define suffering, just like labeling either syndrome or disease expressed in fact the limited level of knowledge in this field.

We appreciate as current and comprehensive the nosological BMS defined by Ţovaru et al. [**4**], in which BMS is considered a syndrome consisting of a complex of clinical symptoms. The importance of the BMS study stems primarily from the impact symptoms have on the quality of life of the patient. The degree of manifestation and impact on the quality of life by BMS varies from the possibility of allowing the patient to carry out normal activities of life to seriously compromised life. The first category of patients, manifest symptoms detached from the syndrome, not reporting a comparison with another, including patients who are “obsessed”, even showing suicidal thoughts. A different behavior of patients is determined not only by how it is perceived at a psychic level, but especially by the intensity of variation of BMS symptoms during the day or over a longer duration of time. The severe cases which are identified during history taking, have distributed a constant intensity of symptoms throughout the day, requiring a patient’s obsessive concern about their health [**10**,**11**].

## General considerations on etiopathogenesis BMS

From an etiopathogenic point of view, the almost universally accepted classification is BMS pain syndrome type in the neuropathic group.

The anatomic substrate that generates excitement is feeling pain and post-processing cortical committed effector responses (motor or secretory), such as the reflex arc. The reflex arc excitation mode of transmission is from the peripheral receptors in the central nervous system and from here to the effector organs.

The following five components are involved in the anatomical pain transmission:

• specific receptor or simply differentiated free terminal nerve

• one or more sensory neurons of the related pathway,

• intra- or extranevraxial reflex center,

• somatic or autonomic efferent pathway and

• muscle or organ effector secretion [**12**].

Information from analyzers plays a key role in the color of affective-emotional pain, memorizing and recalling it subsequently. This decisively contributes to the sensory-sensory patterns, especially if pain is repeated and especially with chronic ones [**13**].

Three types of stimuli can activate pain receptors (nociceptors) in BMS syndrome: mechanical, thermal and chemical. Most of the recent studies admit the existence of a fourth category of nociceptive receptors, namely the multimodal. Their feature is the ability they possess to be activated by all three types of stimuli [**13**,**14**]. Unlike most other sensory receptors of the body, pain receptors adapt little or sometimes not at all. Specific to the nociceptors is the possibility that the excitation painful nerve fibers may gradually increase in intensity as the painful stimulation continues. The relative lack of adaptation of nociceptors is important because the presence of pain keeps the person conscious to the stimulus which violates the tissues while it persists [**15**], resulting in the pain signal or the value of a defense [**16**].

The pathway associated with pain is similar with other ways, such as thermal sensory neurons and synapses containing them.

The interneuronal synapses can produce phenomena of amplification, filtering or sensorimotor inhibition of sensory afferents due to either various ways of connecting with the postsynaptic endings pre- (convergence, divergence, reverb etc.) or the release of chemical differentiated mediators. Of the three functional categories of chemical mediators, some are the main transmitters (excitatory or inhibitory), others put the shoulder to the main proceedings of the first potency (as transmitters) and the latter modulate the intensity of the action (modulators). Among those involved in transmitting excitation, painful as nerve influx, some are excitatory (acetylcholine, substance P, glutamate etc.) or inhibitors (glycine, γ-aminobutyric acid or GABA) [**12**,**16**].

In the mouth, specific to the arch of pain is that it overlaps the one carrying taste. Thus, pain and taste use a common leadership. This gives the hypothetical possibility that in case of change of taste, it becomes the possible cause of nonspecific oral cluster [**17**]. Starting from this hypothetical version and related to the existence of a common path, the medical literature has accepted and defined the theory involving taste arch along with the taste conductor algesia.

The specific of neuropathic pain is that it expresses the existence of damage to the nerve pathways. Being framed as neuropathic pain, BMS results from nerve damage of the nerves serving the gustatory sensation [**18**-**20**].

Recent results of a neurophysiological study suggested that the burning mouth syndrome might be due to nonspecific central neuropathic and/ or peripheral disorder, especially in its primary (essential) form in relation with the ability to identify the cause [**21**].

The expert literature on algia records that in the case of BMS pain associated with other clinical manifestations there are no lesions of oral mucosa, pain does not follow any specific anatomical pathways and there are no known neurological disorders to explain the symptoms. In addition, there are no laboratory characteristic abnormalities [**1**]. Arguments aforementioned imposed the introduction of the name of a painful syndrome to define nonspecific oral symptoms that have no identifiable cause.

For over two decades, BMS etiopathogenesis has been considered multifactorial and multi-pathogenic. Thus, the possible pathogenic mechanisms are formulated as:

a) reduced density of epithelial nerve fibers and a degree of axonal degeneration in patients with BMS lingual biopsies, suggesting a type of trigeminal sensory neuropathy [**22**].

b) the existence of low levels of dopamine in the nigrostriatal pathway as noted in patients with anxiety or other forms of stress [**23**];

c) change transduction of noxious stimuli in the orofacial region;

d) interference and the emergence of changes in the conduct of transmission and modulation of nociceptive information.

Pathogenic changes recorded in c) and d) are invoked to explain the genesis of pain especially in primary BMS.

The possibility of a) and b) to participate are not excluded in the pathogenesis of primary BMS but we have to stress the importance and specificity of the last 2 items.

The hypothesis of an alteration of the gustatory system assumes that gustatory stimuli have an influence on the nociceptive trigeminal inhibitory system. Therefore, a hypogeusia/ ageusia due to peripheral nerve degeneration in patients with BMS could lead to decreased taste sensitivity. At the cortical level, it would result in trigeminal nociceptive central disinhibition, which results in defective processing of information transmitted by a modified perception of pain sensitivity (increase) in the oral region [**21**].

Anatomical connections between taste sensitivity and oral pain argue that the gustatory system damage may be associated with an oral burning sensation or other abnormal type.

In explaining the pathogenesis of pain syndrome in BMS role and importance of land and predisposing factors have been invoked. Thus, BMS could be a feeling at the oral level induced in individuals predisposed to gustatory system damage (“supertasters” - the large number of fungiform lingual papillae). In recent years, we have observed that changes in taste perception and tolerance to pain could be possible causes of burning sensation. Taste is located at the lingual fungiform papillae fundamental level, and in some patients with nonspecific pain syndrome, especially women, there are a large number of these papillae present on the front of the tongue, these patients being referred to as “supertasters” [**24**].

The etiologic factor affecting any taste buds can lead to changes in taste (e.g. infection, trauma, injury, contact sensitivity, nutritional deficiency etc.); deficiency in estrogen at menopause emphasizes sensory changes [**17**].

According to Manole [**3**], the intensity of pain depends on the size of the excitability threshold. The painful receptor density is higher; the excitability threshold is lower, and the pain more intense and unbearable.

The excitation was converted to the feeling at the cortex. The perceived sensation after biting - functional changes of the trigeminal mainly occur as oral burning and as allodynia and xerostomia [**25**].

## Conclusions

In last years, we have observed that changes in taste perception and tolerance to pain could be possible causes of burning sensation. Taste is located at the lingual fundamental fungiform papillae level, and in some patients with nonspecific pain syndrome, especially women, there are a large number of these papillae present on the front of the tongue, these patients being referred to as “supertasters”. Etiologically, any factor affecting the taste buds can lead to changes in taste (e.g. infection, trauma, injury, contact sensitivity, nutritional deficiency, etc.). Menopausal estrogen deficiency accentuates the sensory changes.

Nonspecific pain syndrome is a long-term condition and like all the other chronic pain, is associated with numerous comorbidities. Treatment outcomes are often linked to mental associated disorders such as depression, anxiety, and the presence of chronic pain, afflictions to be considered in the multidisciplinary approach to patients (dentists, neurologists, psychiatrists, etc.).
